# Symbolic Product Superiority in the Neural Salience of Compensatory Consumption Behavior

**DOI:** 10.3389/fpsyg.2020.00838

**Published:** 2020-05-08

**Authors:** Wenjun Yu, Zhongqiang Sun, Zhihui He, Chuyuan Ye, Qingguo Ma

**Affiliations:** ^1^Business School, Ningbo University, Ningbo, China; ^2^Academy of Neuroeconomics and Neuromanagement, Ningbo University, Ningbo, China; ^3^Department of Psychology, Ningbo University, Ningbo, China; ^4^Center of Group Behavior and Social Psychological Service, Ningbo University, Ningbo, China; ^5^School of Management, Zhejiang University, Hangzhou, China

**Keywords:** compensatory consumption, event-related potentials, symbolic product, defeat, purchase intention

## Abstract

To cope with self-threat being induced by personal setbacks in daily life, compensatory consumption, especially on symbolic product, has been found to do valuable help to resolve discrepancies between ideal and actual self-concept. Conforming to symbolic self-completion theory, the current study adopted event-related potentials to explore the objective information processing stages in self-concept-impaired status (the defeat group) on a neural level. The behavioral results replicated previous findings that the defeat group gained stronger purchase intention for symbolic products than utilitarian products. The electrophysiological data demonstrated that perceptual difficulties for products in preliminary stage (N1) were steady among conditions, and after that, information processing separation emerged. In contrast to the individuals with a draw experience, those with a defeat experience raised highly focused attention (P2) and eager expectation (N2) for products, especially for symbolic ones. Meanwhile, symbolic (vs. utilitarian) products also evoked a higher emotional arousal level and slowed the diminishment of involved attentional resource (late positive potential) at late cognitive processing stage. Taken together, the sequential integration of multiple neural indicators contributes to elucidating the processing stages of compensatory consumption behavior.

## Introduction

Reality often falls short of expectations in real life. Humans routinely experience a variety of personal setbacks manifesting as discrepancies between ideal and actual self-concepts (Mandel et al., 2017), including core aspects such as self-identity, authority, intelligence, and perception of affiliation (e.g., Dalton, 2008; Rucker and Galinsky, 2008; Loveland et al., 2010; Lee and Shrum, 2012). The setbacks might result in negative mental states and induce individual motivation to resolve those discrepancies (Tesser, 1988). As an important strategy for coping with self-threats, symbolic consumption can achieve symbolic significance (e.g., status manifestation, conception expression, and class approach) through certain consumption behaviors referred to as compensatory consumption (Gronmo, 1988; Woodruffe-Burton, 1998; Rucker and Galinsky, 2008; Kim and Gal, 2014). As an effective tool to address psychological deficits, the forms of compensatory consumption include compulsive buying (Faber and O’Guinn, 1992), impulsive purchasing (Bayley and Nancarrow, 1998), conspicuous consumption (Roy Chaudhuri et al., 2011), and so forth. A critical shared feature is that consumers seek symbolic rather than utilitarian values from these products or services (Rucker, 2009).

The symbolic self-completion theory (Wicklund and Gollwitzer, 1981) may provide theoretical support for compensatory consumption behavior in which humans tend to cover up or make up for their deficiencies by certain self-symbolized behaviors. More specifically, self-concept may present on several dimensions owing to its complexity; hence, when information on one dimension fails to outline one’s ideal self-image, an individual might seek support from alternative dimensions. One such support that is relatively easy to achieve is symbolic behaviors, among which symbolic product consumption is one of the most common. Compared with traditional utilitarian products, symbolic products are defined as a commodity form that manifests value-related information (Baudrillard, 2016) about its owner, such as wealth, status, habit, and tastes. Consumption behavior specific to symbolic products may play an important role in self-support as one’s possessions are related to self-concept completion and improvement (e.g., Ahuvia, 2005; Oyserman, 2009; Shankar et al., 2009). That is, the possessions, although they are worldly items, can also integrate into the self and further lead self-concept development in a homogeneous direction. Therefore, individuals encountering self-threats commonly cope by repairing their imperfect selves by consuming and exhibiting products, in other words, compensatory consumption. For instance, buying limited-edition products might help in building a wealthy self-concept (Sivanathan and Pettit, 2010).

Empirical behavioral studies in the field of marketing proved this compensatory process. Dalton (2008) found that participants given preset poor performance feedback for an intelligence test preferred to choose products to increase their self-identity (e.g., tee-shirt with an alma mater’s school badge) as well as gift cards from exclusive shops. The reason for these behaviors might be that their failure of the intelligence test induced self-threat, resulting in their consumption of identity-symbolized products to achieve self-worth. Pens were also popular gift options for participants with poor intelligence test performance as they are an intuitive symbol of intelligence (Wang and Wang, 2011). Other studies reported that participants who were told they ranked within the worst 10% in a simple examination tended to pay much more on luxuries or limited-edition collections than those ranking within the best 10%, implying that ranking-symbolized products positively affected their threatened self-concept (Sivanathan and Pettit, 2010). Similarly, products to improve interpersonal relationships received attention from individuals experiencing social exclusion (Mead et al., 2011).

Although previous research showed the ubiquity of the attraction of consumers with impaired self-concept to self-symbolized products, little is known about the underlying psychological processes. People are likely to exhibit behaviors that are inconsistent with their visceral reactions, because some response strategies, exemplified by social desirability bias (Fisher, 1993), might distort their self-reports or subjective ratings. Unlike those output stage data (Yoon et al., 2012), neurophysiological approach helps researchers to observe people’s involuntary reactions without the interference from those strategies that are not expected in the study. Specifically, the current study used event-related potentials (ERPs) to intuitively measure consumer information processing. ERPs have a relatively high temporal accuracy and can be used to investigate neural activities independent of subjective reports (Amodio et al., 2004; Luck, 2005; Ma et al., 2012). A sequential integration of multiple neural indicators would also help to elucidate processing stages throughout a purchase decision in compensatory consumption behavior.

Accordingly, the current study explored the cognitive processes of symbolic or utilitarian products in self-concept-impaired consumers. The impaired status was initially induced by defeat in a multi-round game, followed by a willing-to-buy rating task on symbolic and utilitarian products. Behaviorally, the willing-to-buy task was used to measure purchase intention, while neurologically, we used N1, P2, N2, and late positive potential (LPP) as ERP indicators during information processing.

As a critical component during early perceptual processing, N1 peaked approximately 130–150 ms after stimulus onset, reflecting the individual’s perceptual difficulty with the stimuli (Wang et al., 2018). The following P2, with a peak latency at 100–200 ms after stimulus onset, is sensitive to attentional resource allocation (e.g., Carretié et al., 2001; Huang and Luo, 2006; Jin et al., 2017). Taking emotional stimuli as an example, a study confirmed that P2 amplitude was larger in response to negative stimuli (vs. positive stimuli) that evolutionarily increase the engagement of attentional resources (Carretié et al., 2001). This cognitive function of P2 might contribute to the relationship between purchase intention and variation in attention involvement. The anterior N2 component has also been used to investigate the role of conflict detection (Folstein and Van Petten, 2008; Ma et al., 2010; Liu et al., 2014; Jin et al., 2017; Jing et al., 2019). A mismatch between actual and expected stimulus elicits an N2 component at 200–350 ms, implying cognitive control in decision-making processes. In the late cognitive stages, LPP is an effective indicator of motivation and emotional arousal, occurring 300–500 ms after stimulus onset and lasting hundreds of milliseconds (Nieuwenhuis et al., 2005; Liu et al., 2014; Jin et al., 2017; Jing et al., 2019). As in the marketing literature, LPP amplitude was positively correlated with sustaining attention involvement and purchasing motivation (Ma et al., 2018).

On the basis of the above, we initially speculated that individuals with impaired self-concept would show a stronger subjective purchase intention for symbolic products compared with utilitarian ones, in addition to an analogical performance on a neural level. Neuroscientific techniques allow measurement of underlying psychological stages reflecting the stages of consumer’s decision-making process including perceptual difficulty, early and sustained attention distribution, perceptual conflict, and implicit consuming motivation.

## Materials and Methods

### Participants

Thirty-four graduate and undergraduate students (18 females) with a mean age of 19.94 years (range: 1924 years, *SD* = 1.43) were paid to participate in this experiment. Half of the participants were enrolled in the defeat group (with a defeat experience in the experiment) and the other half in the draw group (with a draw experience in the experiment). All participants had normal or corrected-to-normal vision and provided informed consent before the experiment in compliance with the principles of the Declaration of Helsinki.

### Stimuli

Two types of stimuli were adopted in the experiment. In the time estimation game, we used a 3.0° × 3.0° white square, always positioned in the center of the screen.

The target stimuli consisted of 80 product images, half of which were generally symbolic products, and the other half were generally utilitarian products. In the pretest, we used both behavioral and ERP experiments to ensure the homogeneity of all symbolic or utilitarian products images. The current study finally enrolled symbolic products, including pen, wine, and smart band, and utilitarian products, including ballpoint pen, beer, and clock, with 8–10 items for each type (see example product images in Supplementary Material). All product images were obtained from the Internet, and each measured approximately 5° × 7°, presenting on a gray background (RGB, 80, 80, 80).

### Design and Procedure

Participants were seated in an electrically shielded and sound-attenuated recording chamber at a distance of 70 cm from a 19-inch CRT monitor (with a 100-Hz refresh rate). We used the E-Prime^®^ software to control stimulus presentation and response acquisition. The procedures and study design were approved by the Research Ethics Board of the Academy of Neuroeconomics and Neuromanagement in Ningbo University.

**FIGURE 1:**
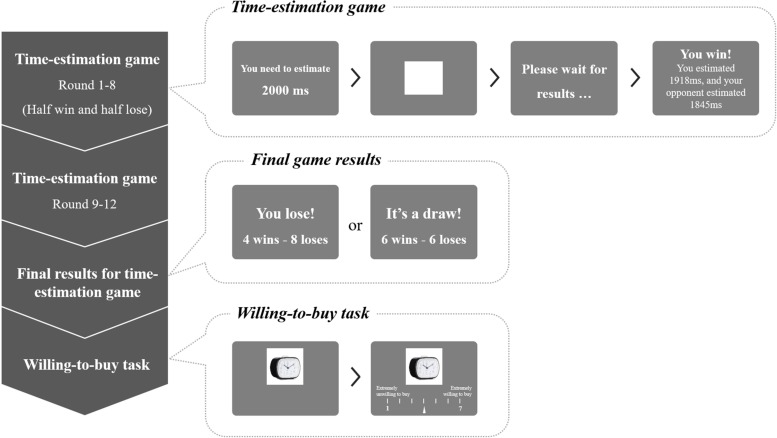
Experimental procedure. The left diagram represents the general process; the top bubble on the right is an example of a round in time-estimation game with 2,000-ms estimation time in a win condition, proceeding from left to right; the middle bubble on the right presents the final results of 12-round time-estimation game; and the bottom bubble on the right is an example of a trial with a utilitarian product, proceeding from left to right.

The participants were provided clear instructions on performing the experimental trials. The experiment comprised two main tasks arranged sequentially, as illustrated in Figure 1.

#### Time-Estimation Game

The first part of the experiment was a time-estimation game (Yu et al., 2018). When the participants entered the laboratory, they were instructed to compete with participants as the opponents in a local area network (LAN)-based game. Their communication was restricted to a greeting when they first met at our laboratory. In the formal experiment, the LAN-based game was an offline game in which the opponent was one of the experimenters in disguise who did not play the game. These manipulations were used to control the participants’ draw or defeat status. This time-estimation game required the participants to estimate an interval as accurately as possible with 12 total rounds. At the beginning of each round, an instruction was presented for 2,000 ms to indicate the interval that needed to be estimated, followed by a white square indicating the start of the game. The participants were instructed to press a button with their right index finger once they thought that the interval had elapsed. The square would then disappear and was replaced by a progress page instructing the participants to wait for the result, which lasted for 2,000–3,000 ms. Feedback was then given visually, informing the participants whether they had won or lost for this single round, along with the estimated times for both the participant and their opponent. This screen indicated the participants’ actual estimated time, whereas the opponents’ estimated time was conditionally controlled by the program. In the win round, the opponents’ absolute value of the estimated time deviation was set to be larger than that of the participants (randomly generated from 50 to 400 ms), whereas in the lose round, the absolute value of the opponents’ estimated time deviation was set to be smaller than that of the participants (randomly generated from 1 to 50 ms). The feedback information was presented for 5 s before the next round began. The interval between rounds was randomly determined from 2,000 to 2,500 ms.

For the first eight rounds, half of the rounds were manipulated as win and half of the rounds as lose, manifesting as a draw status in the first 2/3rd of the game. For the last four rounds, the participants in the draw group would experience two win and two lose rounds, whereas those in the defeat group continuously experienced four lose rounds. At the end of the game, the participant was shown the final game result (being defeated or getting a draw in all 12 rounds) and the numbers of win and lose rounds (4 wins and 8 loses for the defeat group and 6 wins and 6 loses for the draw group).

#### Willing-to-Buy Task

The second part of the experiment was a product willing-to-buy task. At the beginning of each trial, a product image was displayed in the screen center for 1,000 ms, followed by showing a 7-point Likert scale, from 1 (extremely unwilling to buy) to 7 (extremely willing to buy) with an initial pointer at 4. The participants were asked to rate their purchase intention for the displayed product by pressing the left or right buttons to move the pointer to the actual rating and pressing enter to confirm. Their final rating was recorded, with no time limitation for response. The 80 product images were randomly ordered. The task was divided into two blocks separated by a 5-min break.

After all trials had finished, participants were asked if they were aware of the experimental objective. No one answered affirmatively to these questions, and the data of these participants were then used for analysis.

### Electrophysiological Recording and Analyses

Electroencephalogram (EEG) recordings were made at 64 scalp sites by using Ag/AgCl electrodes mounted on an elastic cap. The EEG and electrooculogram (EOG) signals were amplified by a SynAmps2 amplifier (Compumedics NeuroScan, Charlotte, NC, United States) with a sample rate of 500 Hz, using a 0.05- to 100-Hz band-pass filter. The left mastoid reference was used for all recordings, and the data were re-referenced for averaging the left and right mastoid voltages. Vertical EOGs were recorded by one pair of electrodes placed above and below the left eye, and horizontal EOGs were recorded by another pair of electrodes placed 10 mm at the outer canthus of both eyes. Impedances of all inter-electrodes were maintained below 5 kΩ during the experiment.

EEG data were analyzed using NeuroScan 4.3.1 and Curry 8. The initial processing for data was a correction for eye blinks by using a regression procedure. The EEGs were then filtered through a zero-phase shift with a low pass at 30 Hz (24 dB/octave), followed by segmenting into epochs ranging from 200 before to 1,000 ms after the onset of the product image for all conditions, and the epoch was baseline corrected using a 200-ms interval prior to the presentation of the product image. Trials with artifacts exceeding ± 100 μV in amplitude were rejected and excluded from analysis.

To assess N1, P2, and N2, electrode sites in the frontal (F1/Fz/F2) and centrofrontal (FC1/FCz/FC2) regions were selected for further analysis. We pooled electrode data for those two brain regions as a representative site because their patterns were similar. To assess LPP, we chose electrode sites in the centroparietal (CP1/CPz/CP2) and parietal (P1/Pz/P2) regions and pooled the two regions as representative of LPP for the same reason.

On the basis of the averaged waveforms in the present study as well as the results of previous studies, we defined the N1 and N2 peaks as the most negative point within 80–170 and 240–350 ms after product onset, respectively. The P2 peaks were defined as the most positive point within 150–220 ms. The LPP differences between subgroups occurred at 440 ms and lasted about 210 ms; thus, a time window of 440–650 ms after the product onset was used to measure the mean LPP amplitude.

One-sample *t*-tests were first used to examine participants’ subjective purchase intention on products in each category, with a test value of 4 (the median rating in the 7-point Likert scale). With the use of the game result (defeat vs. draw) as the between-subject variable and product category (symbolic vs. utilitarian) as the within-subject variable, two-way repeated-measure analyses of variances (ANOVAs) were used to analyze purchase intention; amplitudes of product-onset N1, P2, and N2; and mean LPP amplitude during the time window of interest.

## Results

### Behavioral Results

**FIGURE 2:**
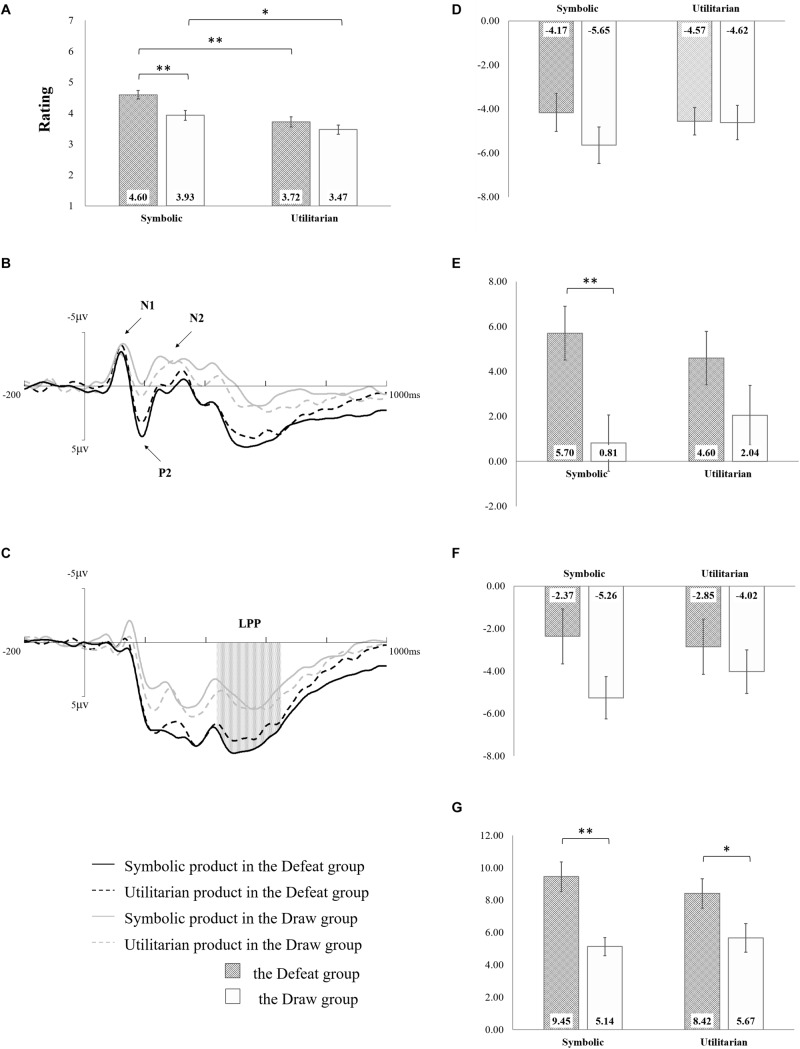
Experiment results. **(A)** Behavioral results. **(B)** Averaged F1, F2, Fz, FC1, FC2, and FCz waveforms. **(C)** Averaged CP1, CP2, CPz, P1, P2, and Pz waveforms. **(D)** Mean peak N1 amplitude. **(E)** Peak mean P2 amplitude. **(F)** Mean peak N2 amplitude. **(G)** Mean late positive potential (LPP) amplitude. The asterisks represent significant differences (^∗^0.01 < *p* < 0.05, ^∗∗^*p* < 0.01) between two corresponding conditions; the error bars represent one SEM; and the absent label between two conditions means non-significant difference.

Only the rating for symbolic products in the defeat group exceeded the objective medium willing-to-buy rating; moreover, the subjective willing-to-buy ratings in both product categories in the defeat group were higher than those in the draw group (Figure 2A). Confirming this observation, one-sample *t*-tests revealed a significantly higher willing-to-buy rating for the symbolic product in the defeat group [*M* = 4.60, *SE* = 0.14, *t*(16) = 4.11, *p* = 0.001], and, in contrast, a lower interest in the utilitarian product in the draw group [*M* = 3.47, *SE* = 0.15, *t*(16) = -3.60, *p* = 0.002]. No significance was observed for the utilitarian product in the defeat group (*M* = 3.72, *SE* = 0.16, *p* = 0.09) or the symbolic product in the draw group (*M* = 3.93, *SE* = 0.16, *p* = 0.67).

The 2(game result) × 2(category) ANOVA showed the main effects of both game result [*F*(1,32) = 5.44, *p* = 0.026, η_*p*_^2^ = 0.145] and category [*F*(1,32) = 54.0, *p* < 0.001, η_*p*_^2^ = 0.628], in addition to the interaction between the two variables [*F*(1,32) = 5.16, *p* = 0.03, η_*p*_^2^ = 0.139]. The defeat group (*M* = 4.16, *SE* = 0.14) rated higher than the draw group (*M* = 3.70, *SE* = 0.14); moreover, symbolic products (*M* = 4.26, *SE* = 0.11) had a larger purchase intention than did utilitarian products (*M* = 3.59, *SE* = 0.11). Moreover, the increment from utilitarian to symbolic products was larger in the defeat group (*d* = 0.88) than that in the draw group (*d* = 0.46).

### Event-Related Potential Results

#### N1

As shown in Figures 2B,D, the 2(game-result) × 2(category) ANOVA for N1 peak amplitude showed a marginally significant interaction between two variables [*F*(1,32) = 3.60, *p* = 0.067, η_*p*_^2^ = 0.101]. The increment of N1 (negative polarity: a smaller amplitude value indicates a higher N1 amplitude) from utilitarian to symbolic products tended to be smaller in the defeat group (*d* = -0.4) than that in the draw group (*d* = 1.03). No significant main effect was observed for game result [*F*(1,32) = 0.55, *p* = 0.465, η_*p*_^2^ = 0.017] or category, [*F*(1,32) = 0.69, *p* = 0.411, η_*p*_^2^ = 0.021].

#### P2

The P2 results are shown in Figures 2B,E. The 2(game-result) × 2(category) ANOVA for peak amplitude of P2 revealed a significant main effect of game result [*F*(1,32) = 4.68, *p* = 0.038, η_*p*_^2^ = 0.128] and an interaction effect between two variables [*F*(1,32) = 10.63, *p* = 0.003, η_*p*_^2^ = 0.249] in which the defeat group (*M* = 5.15, *SE* = 0.83) had a larger P2 amplitude than the draw group (*M* = 1.43, *SE* = 0.91), whereas the P2 increment (positive polarity: larger amplitude indicated increased P2 amplitude) from utilitarian to symbolic products was larger in the defeat group (*d* = 1.10) than in the draw group (*d* = -1.23). However, the main effect of category was not revealed [*F*(1,32) = 0.03, *p* = 0.855, η_*p*_^2^ = 0.001].

#### N2

The peak amplitudes of N2 showed similar patterns to those observed for N1 (Figures 2B,F). The 2(game-result) × 2(category) ANOVA for peak amplitude of N2 showed a significant interaction between two variables [*F*(1,32) = 6.11, *p* = 0.019, η_*p*_^2^ = 0.160], with a more negative N2 increment (negative polarity: more negative amplitude value indicated increased N2 amplitude) from utilitarian to symbolic products in the draw group (*d* = -1.24) than in the defeat group (*d* = 0.48). The main effects of game result [*F*(1,32) = 1.59, *p* = 0.216, η_*p*_^2^ = 0.047] and category [*F*(1,32) = 1.15, *p* = 0.292, η_*p*_^2^ = 0.035] were not statistically significant.

#### Late Positive Potential

The 2(game-result) × 2(category) ANOVA results for the mean LPP amplitudes at 440–650 ms (Figures 2C,G) indicated that the defeat group showed a larger amplitude (*M* = 8.94, *SE* = 0.80) than the draw group (*M* = 5.41, *SE* = 0.80) [*F*(1,32) = 9.84, *p* = 0.004, η_*p*_^2^ = 0.24]. Meanwhile, the significant interaction between game result and category [*F*(1,32) = 5.73, *p* = 0.023, η_*p*_^2^ = 0.152] indicated that the LPP increment (positive polarity) from utilitarian to symbolic products was larger in the defeat group (*d* = 1.03) than in the draw group (*d* = -0.53). The main effect of category was not obvious [*F*(1,32) = 0.59, *p* = 0.450, η_*p*_^2^ = 0.018].

## Discussion

This study assessed purchase intention for symbolic products in participants with impaired self-concept and explored the underlying information processing stages in these participants. In line with previous studies, the behavioral results showed a higher purchase intention for participants in an incidental defeated status compared with that in participants with a draw status; furthermore, as anticipated, the effect was increased for symbolic products. At the neural level, the early-elicited N1s were similar among conditions. Two ERP components with positive polarity, P2, and LPP were higher in the defeat group than in the draw group; and P2, N2, and LPP showed separation between symbolic and utilitarian products, with as stronger changes in neural activity for symbolic products.

The behavioral results confirmed our hypothesis that defeat status increased purchase intention (vs. draw status), especially for symbolic products. On the basis of the symbolic self-completion theory and previous findings (e.g., Dalton, 2008; Sivanathan and Pettit, 2010; Wang and Wang, 2011), we inferred that defeat in the time-estimation task would induce self-doubt and a discrepancy between actual and ideal self-concepts. To effectively make up for this deficiency, individuals seek products with corresponding self-support values from alternative self-symbolizing behaviors (Wicklund and Gollwitzer, 1981), and a strong purchase intention for symbolic (vs. utilitarian) products emerges. In addition, the main effect of category replicates those previously reported (Elliott and Wattanasuwan, 1998), implying the overall existence of symbolic consumption behavior.

The ERP results provided more concise interpretations of information processing in compensatory consumption. At the preliminary stage, N1 showed similar patterns for all product images, suggesting a stable perceptual difficulty in perceptual behavior control (Wang et al., 2018). In other words, regardless of competition outcome and product category, this factor was homogeneous for those products in the participants’ evaluation of purchasing disadvantage, including perceptual emergency and purchase conveniences.

The following P2, as a robust reflection of attention involvement (Carretié et al., 2001; Huang and Luo, 2006; Jin et al., 2017), was higher in the defeat group than that in the draw group. The difference in P2 amplitude was most likely due to a highly concentrated allocation of attentional resources in individuals with impaired self-concept. According to product category, the draw group tended to show higher rational engagement with utilitarian attributes, but the situation changed for participants with impaired self-concept. The defeat group showed predominant attentional resource distribution for symbolic products; in other words, when the participants were defeated, they raised more concerns about symbolic attributes (vs. utilitarian attributes).

During mid-stage information processing, N2 is an index of the similarity between actual and expected stimuli (Ma et al., 2010). The interaction effect showed a more negative N2 for symbolic products compared with utilitarian products in the draw group, whereas the N2 amplitude was sharply decreased in the defeat group. A self-concept discrepancy may motivate a participant’s need for self-support, which is the aim of symbolic products. The close similarity in symbolic attributes between the actual and expected products led to a relatively weak perceptual conflict in purchase intention evaluation.

Research in consumer psychology demonstrated a probable relationship between N2 and perceived risk because risk and conflict are interrelated (Folstein and Van Petten, 2008; Spapé et al., 2011; Larson et al., 2012; Ma et al., 2015; Wang et al., 2016). Compared with products with high review ratings, those with low ratings evoked greater perceived decision risk, resulting in a larger N2 amplitude. In the current study, the apparent symbolic attributes in symbolic products reduced the perceived risk; thus, the participants’ inhibitions were weakened; more simply, they were more willing to make a purchase decision.

Concerning LPP in the late processing stage, the current study demonstrated both a significant main effect of game result and interaction between variables. An enhanced LPP within a time window of interest indicates a sustained involvement of cognitive resources and high emotional arousal on motivationally salient stimuli (Ma et al., 2018; Wang et al., 2018). Thus, the data from the present study suggested that defeat status (vs. draw) slowed the diminishment of involved attentional resources for displayed products; meanwhile, emotion arousal would be intense, implying a willingness to make purchase decisions on those products. This effect would be greater for symbolic products because their possession could improve the self-concept completeness.

Although the perceptual difficulty for products in the preliminary stages was constant among all conditions, three ERP deflections—P2, N2, and LPP—were modulated by the game results and product categories and showed similar patterns. Previous studies also reported the priority of symbolic products of various dimensions in self-concept-impaired situations (Dalton, 2008; Rucker, 2009); however, this is not the only influence on consumption behavior from the compensatory need. Current findings further revealed multi-stage cognitive processing for compensatory consumption behavior: high concern and eager anticipation for symbolic products increase to meet compensatory demand; in turn, those products help evoke higher emotional arousal and sustain attention, finally affecting the purchase intention.

The findings of the present study provide insight for sellers and demonstrate that consumption can be biased by incidental mental status. Along with the heavy life and social stresses, consumers are interested in symbolic products that highlight their personal values in the context of social status, intelligence, lifestyle, and so forth, because the possession of such products seems to be the easiest way for them to reconstruct their self-identity. Thus, subtle and moderate hints in advertisements to motivate a compensatory status might trigger consumer resonance. In coordination with an emphasis on its symbolic significance, consumers may pay increased attention to the product and further raise their purchase intention. In addition, the neuroscientific approach in current study theoretically contribute to revealing some detailed phases of cognitive processing underlying the external purchase preference on symbolic products in compensatory consumption, as well as avoiding the influences of confounding factors such as social desirability to a large extent. Furthermore, considering that the comparison between the defeat and draw groups in current study separated the game results from competition, the overall influence of the competition experience and results on subsequent consumption behaviors is also a rich topic in future studies.

## Data Availability Statement

The datasets generated for this study are available on request to the corresponding author.

## Ethics Statement

The studies involving human participants were reviewed and approved by the Research Ethics Board of the Academy of Neuroeconomics and Neuromanagement in Ningbo University. The patients/participants provided their written informed consent to participate in this study.

## Author Contributions

WY and ZS contributed to the conception and design of the study, performed the statistical analysis. ZH and CY performed the experiments. WY wrote the first draft of the manuscript. QM reviewed and improved the manuscript. All authors read and approved the submitted version.

## Conflict of Interest

The authors declare that the research was conducted in the absence of any commercial or financial relationships that could be construed as a potential conflict of interest.
