# Supplementary figures and images for: Symbolic Product Superiority in the Neural Salience of Compensatory Consumption Behavior
Source: Front Psychol. 2020 May 8;11:838. doi: 10.3389/fpsyg.2020.00838 (PMC7225264; doi:10.3389/fpsyg.2020.00838)

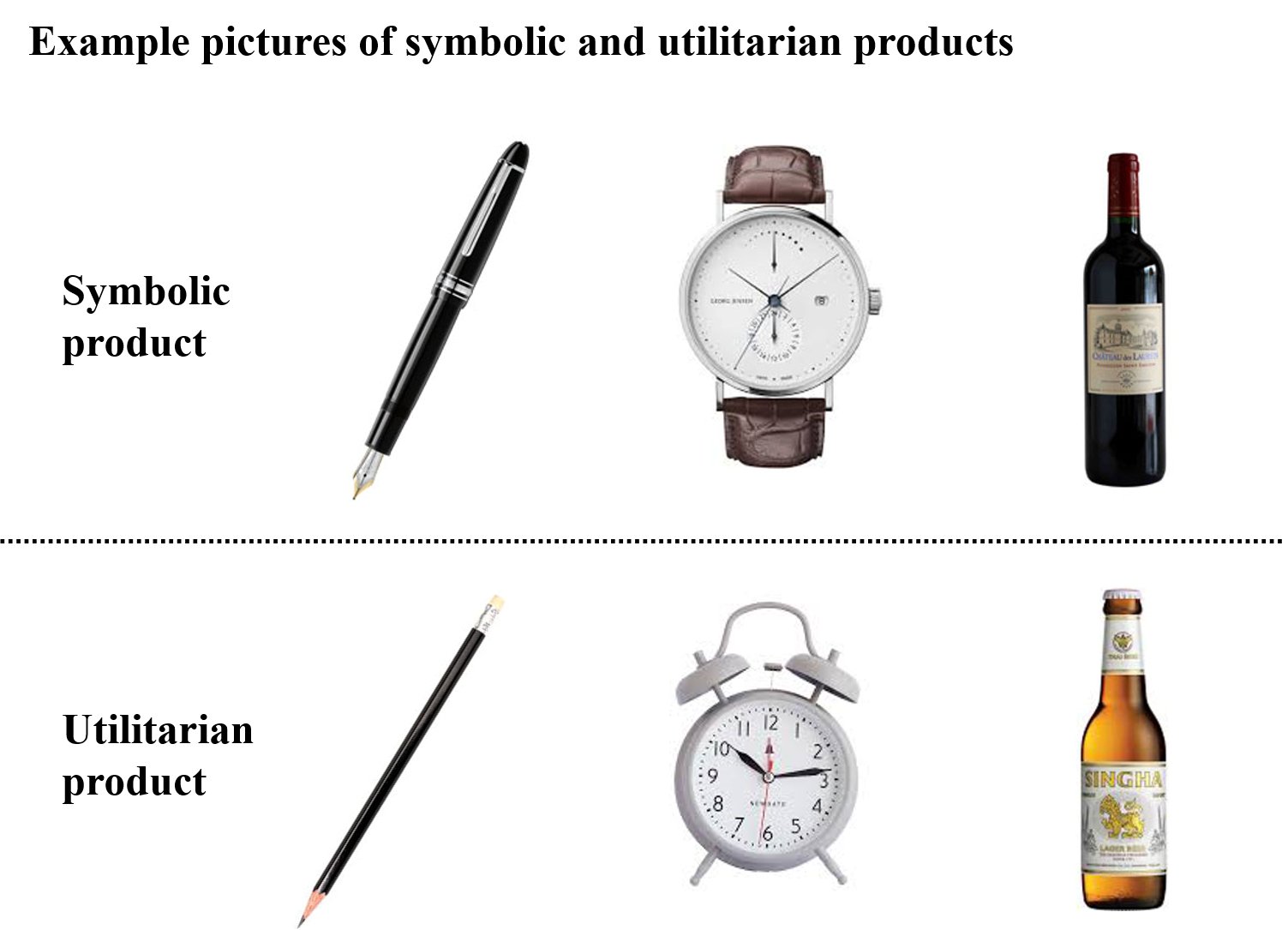

Supplement: Supplementary file 1 [file Image_1.TIF]
